# Clean production and characterization of nanobubbles using laser energy deposition

**DOI:** 10.1016/j.ultsonch.2023.106321

**Published:** 2023-02-09

**Authors:** Juan Manuel Rosselló, Claus-Dieter Ohl

**Affiliations:** a Otto von Guericke University Magdeburg, Institute of Physics, Universitätsplatz 2, 39106 Magdeburg, Germany; b Faculty of Mechanical Engineering, University of Ljubljana, Askerceva 6, 1000 Ljubljana, Slovenia

**Keywords:** Laser-induced bubbles, Nanobubbles, Shockwave, Gas diffusion, Shadowgraph microscopy

## Abstract

We have demonstrated the production of laser bulk nanobubbles (BNB) with ambient radii typically below 500 nm. The gaseous nature of the nanometric objects was confirmed by a focused acoustic pulse that expands the gas cavities to a size that can be visualized with optical microscopy. The BNBs were produced on demand by a collimated high-energy laser pulse in a “clean” way, meaning that no solid particles or drops were introduced in the sample by the generation method. This is a clear advantage relative to the other standard BNB production techniques. Accordingly, the role of nanometric particles in laser bubble production is discussed. The characteristics of the nanobubbles were evaluated with two alternative methods. The first one measures the response of the BNBs to acoustic pulses of increasing amplitude to estimate their rest radius through the calculation of the dynamics Blake threshold. The second one is based on the bubble dissolution dynamics and the correlation of the bubble’s lifetime with its initial size. The high reproducibility of the present system in combination with automated data acquisition and analysis constitutes a sound tool for studying the effects of the liquid and gas properties on the stability of the BNBs solution.

## 1 Introduction

Bulk nanobubbles (BNB) are gas bubbles whose rest radius is sufficiently small to form a stable emulsion and remain in Brownian motion for longer periods than micrometric bubbles [1]. The alleged long life of BNB is still a matter of debate in the scientific community. This is because, according to classical diffusion theory, microscopic spherical bubbles should rapidly dissolve due to the Laplace pressure [2], [3]. As the Laplace pressure increases rapidly with the smaller size of the bubble the term *Laplace catastrophe* was coined [1], [4]. This simple model contradicts the numerous experimental findings of bulk nanobubbles remaining suspended in liquids for many days [5], [6], [7], [8] or even months [9] reported in the last two decades. The particular properties of BNBs offer a diverse range of potential applications that fueled the interest in understanding and generating nanobubbles not only in academia but also in the industrial sectors. These little cavities are believed to have an impact in areas such as waste treatment, cleaning, and purification technologies [10], [11], [12], [13], [14], in biomedicine as for drug delivery [15], [16], [17] or as contrast agents [18], [19], food processing [20], sonochemistry [21], [4], [22], and promote the delivery of oxygen to marine aquatic life and livestock in fish farms [23].

The BNBs can be generated by a variety of methods. Probably the most common are the ones using some kind of mechanical shear stress to induce cavitation [24], [25], [6] or ultrasound irradiation of the liquid samples [9], [26], [27], [28], [29], [7]. Both processes drive the bubbles into a continuous mixing and fragmentation into smaller pieces. Other less frequent alternatives include repeated and controlled liquid pressure variations [30], [31], [5], [32], hydrodynamic cavitation in a Venturi-like nozzle [33], [8], electrolysis [34], or chemical reactions like the one occurring when mixing alcohols and water [35], [36], [37].

One common factor of all these methods is that they are prone to introduce contamination into the liquid during nanobubble production. This pollution may originate from solid particles or drops of an insoluble substance like for example oil. Specifically, the mechanical or acoustic driving methods along with the ones employing hydrodynamic cavitation can introduce particles produced for instance by cavitation erosion of both the movable parts and the container/tubing [38], [39]. Additionally, injection of little drops from lubricants or fragments of the seals commonly found in pistons and pumps may occur. The method based on electrolysis to produce the gas cavities is susceptible to the disintegration of the electrodes or the isolation materials. At last, alcohols and other solvent exchange methods are known to induce the production of little oil drops through the Ouzo effect [40], [36], [39], [37].

Besides the many ways that BNBs preparations could get contaminated there is another important factor that might contribute to the controversy surrounding BNB research, i.e., the way the bubbles are detected and quantified. The standard methods used to measure the size distribution of BNBs are given by some variation of dynamic light scattering (DLS) or laser particle tracking [34], [35], [41], [5]. Even if both techniques are widely adopted, probably because they are relatively simple to implement, they have a major issue when applied to BNB observation, they can not distinguish between solid particles, bubbles, and drops [24], [42], [43], [37]. Consequently, if the nanobubble sample solution gets polluted with spurious material during the bubble production, the impurities might be accounted as nanobubbles leading to incorrect or biased conclusions. The absence of confirmatory unambiguous evidence of the long-term stability of bulk nanobubbles put in question their very existence [40], [9], [36], [39], [32], [44].

The nucleation of cavitation bubbles after a liquid is exposed to a high-power laser beam has been known for many decades and is still under study [45], [46], [47], [48], [49]. In the literature, the observation of small gaseous cavitation nuclei on the sides of a tightly focused laser beam is referred to as “secondary” bubbles [50], [51], [52]. In those cases, the presence of the tiny bubbles is revealed by the pressure fluctuations generated by a bigger cavitation bubble produced at the focal spot of the laser (through the dielectric breakdown). Closely related, the generation of multiple and homogeneously distributed cavitation nuclei by using a non-focused high-power laser beam was reported in a sucrose solution supersaturated with CO_2_ [53], [54]. A similar bubble structure was observed when a laser was fired into a phosphoric acid aqueous solution while applying a continuous acoustic field to lower the cavitation threshold [55]. Very recently, the formation of nanobubbles in absence of fluctuating pressure fields was demonstrated by Rosselló and Ohl in Ref. [56], when a volume of water was illuminated by a pulsed collimated laser beam.

As it is mentioned in the previous paragraphs, the BNB’s long-term stability is currently under intense scrutiny. In the last few years, different stabilizing agents and methods have been proposed to address this aspect. Its relevance resides not only in developing experimental methods or industrial processes *per se* but also to shed some light on the underlying mechanisms behind the bubble diffusive and thermodynamic equilibrium [57], [44], [58]. Such insights could lead to establishing a new theoretical basis. Of the many hypotheses proposed to explain BNB stability, there are two that stand out. The first one assumes the formation of an electronic double layer around the nanobubble that prevents its shrinking and total extinction due to the repulsion of the charges on the bubble interface sharing the same polarity [9], [57], [59]. Another hypothesis suggests that gas diffusion could be partially blocked by the deposition of nanoparticles on the NB surface [60], [21]. Among the many physical parameters whose effect on the BNB stability was analyzed and accounted for one can find: dissolved–gas concentration [27], the temperature of the liquid [29], ionic concentration through the addition of salts, basis or acids [61], [62], [9], [30], [5], [8], [7], liquid surface tension through the addition of surfactants [9], [63] or lipids to coat the bubbles [19].

In this work, we propose an alternative method for BNB generation and characterization that overcomes all the issues discussed above. Here, the bubbles are produced by the deposition of laser energy on spurious particles already present in the working liquid. This optical bulk nanobubble generation method has many advantages regarding the “purity” of the nanobubble preparation. As the laser-induced technique does not require moving parts in contact with collapsing bubbles, like for instance the usual mechanical agitation methods, the final sample is unlikely to get contaminated. Additionally, we implement an acoustically probed bubble detection method, which consists in passing a rarefaction wave through the volume where the bubbles were formerly “seeded” by the laser beam to expand them into a visible size [56]. This represents a significant improvement over the standard DLS and optical characterization methods since the acoustic probe reveals the gas nature of the identified objects without ambiguities.

## 2 Experimental method

**Fig. 1:**
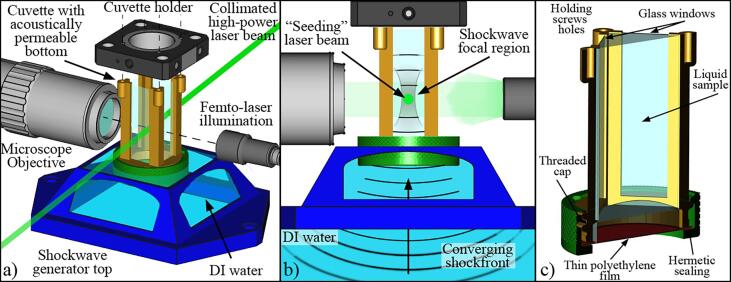
Experimental setup. a) General view. A tailored cuvette with a side of 1.5 cm is held from its top from four points and is partially submerged in the DI water, which transmits the pressure wave generated by the lithotripter. The bubbles are observed from a direction perpendicular to the seeding laser beam. b) Shockwave focusing. The seeding laser beam position matches the pressure focal region in the test cuvette. The pyramidal shape of the lithotripter water reservoir top was designed to allow the microscope objective to get close enough to the cuvette in order to have its focal plane where the nanobubbles are generated by the laser beam. The illumination in the shadowgraph images is performed with a green pulsed laser light transported with an optical fiber. c) Cuvette design. The acoustically permeable bottom of the cuvette is a polyethylene film that lets the shockwave pass through the liquid column without significant attenuation or distortion. The walls of the cuvette are made from four glass windows, while the bottom is sealed with a threaded cap and a flat rubber O-ring.

The experimental method can be summarized as follows: A small liquid volume (∼10 ml) contained in a tailored 3D-printed cuvette is illuminated with a high-power pulse laser to produce bulk nanobubbles. These bubbles are initially not resolvable through microscopy imaging, as their size is below the optical resolution. After a time Δt, a piezoelectric shock wave generator (a modified lithotripter *Piezolith 100, Richard Wolf GmbH*, Knittlingen, Germany) focuses a shock followed by a tension wave through the cuvette. The tensile cycle of the wave expands the nanobubbles such that they become visible. The details of the experimental setup are shown in Fig. 1. Fig. 1(a) depicts the tailor-made 3D-printed cuvette of 5 cm height with a square cross-section (1.5 cm width), whose bottom is a thin polyethylene film. This ensures that the sample liquid in the cuvette is separated from the large and contaminant-prone basin of the shock wave generator. To ensure good acoustic contact, the cuvette is partially submerged 5 mm in the shock wave basin filled with DI water. The cuvette with the liquid sample is held from its top placed such that it makes contact with the basin’s water, i.e., along the focusing path of the shock/tension wave, see Fig. 1(b). The walls of the cuvette are made from four glass windows, while the bottom is sealed with a threaded cap and a rubber O-ring. The cuvette’s bottom wall is acoustically transparent, thus the wave passes without significant attenuation or distortion to expand the nanobubbles produced by the laser pulse, see Fig. 1(c).

The shockwave is generated by driving in parallel many individual piezoelectric ceramics arranged on a spherical cap with a high-voltage pulse. Geometric and non-linear focusing leads to the steepening of the positive pressure transient into a shock front at the focus region. The location of maximum pressure is approximately at the geometric center of the spherical cap. The high pressure is trailed by a negative pressure caused by diffraction waves from the limited aperture[64]. As a result, a cigar-shaped region with a diameter of 3 mm and length of about 8 mm is formed, where the negative pressure phase drops by half of the maximum pressure of the compression phase (positive pressure). At the focus, a bipolar pressure wave with a positive amplitude of ∼34 MPa, and a negative pressure of approximately −16.5 MPa is obtained in the rarefaction phase. We use this device due to its high repeatability between runs, leading to almost indistinguishable traces of the waves from one run to the following. The shockwaves are characterized with a fiber optic hydrophone *Onda HFO-690* (100 μm of spatial resolution) connected to an oscilloscope *Teledyne WavePro 404HD-MS* with a sampling rate of 20 GSa/s and an analog bandwidth of 4 GHz. For more details on the shock/tension wave see Appendix A.

The nanobubbles are nucleated with the help of a green Q-switched Nd:YAG laser (*Litron Nano T-250–10*, λ = 532  nm, FWHM pulse duration of 7 ns). The laser beam passes perpendicular through the acoustic focal volume of the cuvette. The diameter of the beam with an approximately Gaussian intensity profile is first reduced to ∼2 mm with a beam collimator, and then the central part of the beam is extracted with a circular metallic aperture with a diameter of 800 μm. The energy of the collimated laser beam is measured with a pyroelectric sensor (*ES120C, Thorlabs*) and read out with an energy meter (*PM100D, Thorlabs*). The pulse energy of the 800 μm wide beam can be adjusted between 0 and 12 mJ. At a wavelength of 532 nm, the laser pulse passes through the cuvette without suffering a significant loss in energy.

Considering the extremely fast dynamics of both the nanobubbles production and the shock/tension wave, high-speed video recordings are essential to capture the acoustic response of the BNB. Here, we used a camera with a frame rate of 5 Mfps (*XPV-X2, Shimadzu*) which complemented with femtosecond laser illumination (*FemtoLux 3, Ekspla*, λ = 515 nm, pulse duration of 230 fs) ensures sharp, non-motion blurred, and speckle-free images [56]. The frequency of the ultra-short illumination pulses matches that of the camera frame rate. The images were acquired by combining optical microscopy and shadowgraphy. Therefore, the light is coupled into and transported with a 600 μm optical fiber and expanded with a coupling lens (*F230SMA-A, Thorlabs*) for uniform illumination. The necessary large magnification is achieved with a long-distance microscope objective (*Edmund Optics*) with 5X, 10X, 20X, or 50X in line with a second macro lens (*f2.8 macro lens, LAOWA*) with a variable magnification of up to 2X. This allowed us to reach optical resolutions ranging from 7 μm/px to a minimum of 217 nm/px. As shown in Fig. 1, the bubbles are observed from a direction perpendicular to the seeding laser beam and illuminated by the green pulsed laser light.

### 2.1 Automated measurements and image analysis

In the experiments, the shock wave generator, the pulse laser, the oscilloscope, and the camera can be triggered with little jitter. The high repeatability of the pressure wave was necessary to produce automated measurements and obtain sufficient data for statistical analysis. A delay/pulse generator (*Model 9520, Quantum Composers*) controlled with a microcontroller (*Arduino Nano*) sets the timing and synchronization with the pulse train of the light source, which in turn after a suitable delay, triggers all the other equipment. It is important to note that between runs, a waiting time of 30 s or 60 s is deemed necessary such that bubbles have sufficient time to dissolve and/or the flow created dies out.

The high-speed videos captured during the experiments are then analyzed using in-house developed image processing routines. Those characterize the bubble number and their distribution by means of a bubble recognition algorithm based on the Hough transform [65]. The parameters of the detection algorithm are manually adjusted in order to minimize the difference in the area occupied by the bubbles and the area of the detected bubbles in the images. The uncertainty linked to this detection method is typically below 2% of the detected bubble number for most of the cases, but it rapidly increases to 4% when the images present an excessive overlapping of bubbles, i.e., when dense bubble populations are produced.

## 3 Experimental results

The experiments are carried out to explore the effect of the physical parameters of the laser pulse energy and the amplitude of the bi-polar wave on the number of nanobubbles nucleated. Also, the dissolution of the nanobubbles is analyzed by counting how many of them remain in the liquid after some time interval of generation (Δt). For most of the experiments, we use milli-Q water that is additionally filtered by passing through a 50 nm pore size (*EnviroFalk GmbH BMK1EHV1*, 0.1–10 MΩ cm). When treated this way, the water is termed “nanopore water”.

The ability of impurities to nucleate nanobubbles was studied with the highest magnification achievable by our optical setup (i.e., 217 ± 5 nm/px). In some of the experiments, impurities were deliberately added to the nanopore water and their importance in the nucleation of bubbles is discussed. This aspect is further studied in Appendix B.

### 3.1 Bubble nucleation process

**Fig. 2:**
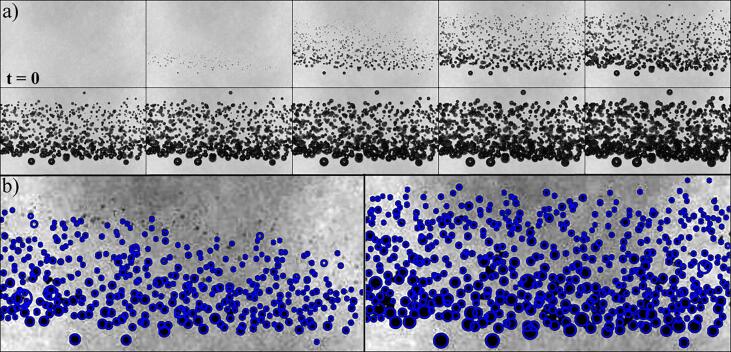
Detection of laser seeded nanobubbles. The energy of the laser pulse is 9.5±0.3 mJ and the negative pressure amplitude in the bi-polar wave is -15.9±0.3 MPa. a) The image sequence shows an example of how the passage of a rarefaction wave (coming from the bottom of the frame) induces the expansion of the bubbles previously nucleated by the laser pulse (i.e., at *t* = 0), making them visible only within the limits of the light beam. The time between frames is 200 ns. The optical resolution here is 6 μm/px and the frame width is 1.5 mm. b) Example of the bubble detection algorithm from two consecutive frames of the video. The bubbles accounted for by the recognition algorithm are encircled in blue. The error associated with this method is below 3%, but it can rapidly grow once they overlap.

We start with Fig. 2(a) presenting selected frames from a high-speed video recording using nanopore water, and with Fig. 2(b) demonstrating the implementation of the bubble detection algorithm. Fig. 2(a) shows ten frames in sequence, taken with an inter-frame interval of 200 ns, as an example of the growth of the initially invisible laser-induced nanobubbles into visible cavitation bubbles as the rarefaction wave travels from the bottom to the top of the frame. Cavities are only observed within the region illuminated by the laser beam, ruling out acoustic cavitation as the bubble nucleation mechanism. The detected bubbles using the above-mentioned image processing routines are shown in Fig. 2(b).

**Fig. 3:**
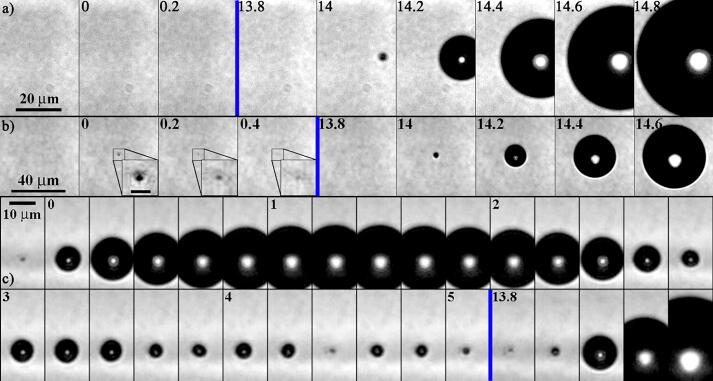
Detailed view of the three scenarios observed during the laser nucleation process captured with higher spatial and temporal resolution. a) Nucleation of a nanobubble with a size bellow a minimum resolution of 217 nm/px. b) In this case the bubble becomes visible for an instant after the laser shot but then shrinks to a size below the optical resolution. The scale bar in the insets represents 5 μm. c) Here the bubble is clearly seeded on a solid particle. The resolution on b) and c) is 408 nm/px. The number in the upper left of the frames indicates the elapsed time in microseconds following the laser pulse.

To answer the question of what causes the nucleation of bubbles, we recorded at our highest magnification the region of interest from the time of the laser pulse till the passage of the rarefaction wave. Overall, the bubble nucleation process could be categorized into two different scenarios, see Fig. 3(a)–(c). Case 1 in Fig. 3(a): in about 92% of the cases, the bubble is not visible during and following the laser pulse seeding, and the bubble only nucleates with the passage of the rarefaction wave. In these cases, no objects such as pre-existing particles or bubbles are visible at the time of the laser pulse t=0. Hence, the bubbles grow from nanobubbles with sizes below the optical resolution only when the negative pressure of the bi-polar wave (arriving at *t* = 13.8 μs in Fig. 3) expands them. Case 2 in Fig. 3(b): around 8% of the bubbles become already visible during the seeding process for several hundreds of nanoseconds and shrink to an invisible size afterward. Then during the rarefaction wave passage, a bubble expands at the very location where the initial expansion was observed now to a much larger size. A possible explanation for the initial expansion could be the vaporization of liquid through absorption and heating of a suspended particle present in the nanopore water that is not resolved optically [54]. This mechanism of particle-based bubble nucleation resembles the heating of gold nanoparticles through plasmonic resonance [66], [67], [68], [69]. While we do not expect gold nanoparticles to be present, we can’t rule out that the nanopore water may still contain mineral-based nanoparticles that are smaller than the filter pores. Yet the heterogeneous thermal heating of solid particles of sizes below 50 nm is only a hypothesis, however, this would predict that larger particles nucleate larger bubbles. To test this idea we repeated the experiment with regular tap water. In this unfiltered water, many more bubbles than in nanopore water are generated [53], [54]. An example of a single resolved particle nucleating a bubble following the laser pulse is exemplary shown in Fig. 3(c). The particle of unknown origin is visible in the first frame. At t=0, a significantly bigger bubble is nucleated as compared to Fig. 3(b). The missing plasma emission suggests that linear heating is nucleating the bubble and not an optical breakdown [48], [70]. The laser-induced bubble expands, collapses, and shows a couple of rebounds before it is expanded into a very large cavitation bubble once the rarefaction wave traverses the frame from *t* = 13.8 μs on.

### 3.2 Bubble count for different energies of the laser pulse

**Fig. 4:**
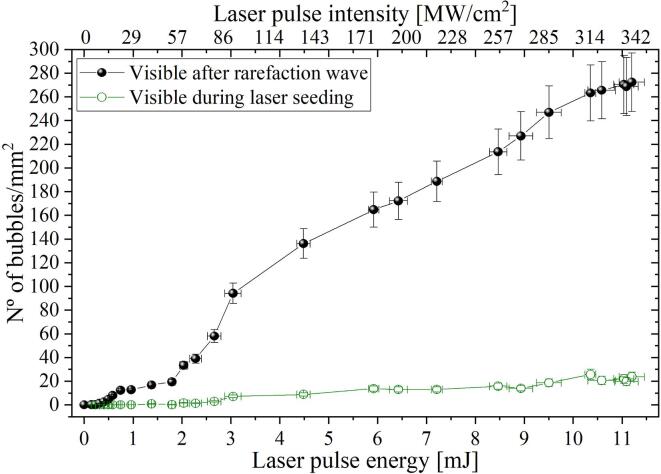
Bubble number density as a function of the laser energy and the laser intensity. The negative pressure amplitude in the bi-polar wave was set to -15.8±0.2 MPa. For this pressure, the onset of the cavitation is approximately 60 MW/cm^2^ (i.e., 2.2 mJ). Here, the laser was fired 10 μs prior to the shockwave arrival. No bubbles were observed in the control cases where the laser pulse was not fired. The resolution of the analyzed images is 5.5 μ/px. Each symbol is the average of 10 measurements. The error in the bubble density was propagated considering a nominal uncertainty of 3% in the bubble number and 0.05 mm^2^ in the area they occupy.

In this section, we explore the threshold laser intensity for the production of bulk nanobubbles by varying the laser pulse energy for a fixed beam diameter. The laser energy is varied between 0 and 12 mJ and the resulting number of expanded bubbles after the passage of the rarefaction wave is counted. Fig. 4 depicts the result for a shock wave with a negative pressure amplitude of -15.8±0.2 MPa. For this pressure, we observe a rapid growth in the bubble density for laser energies higher than 2 mJ, which corresponds to a laser intensity of around 58 MW/cm^2^. This value is more than two orders of magnitude smaller than the typical cavitation threshold value reported in the literature for multiphoton ionization of water at a wavelength of 532 nm, i.e., $I_{\mathrm{thres}}=30$ GW/cm^2^ [46], [47], [71]. It is worth noticing that having the laser intensity below the optical breakdown threshold supports the hypothesis of heated nanoparticles as the mechanism for bubble generation.

For low pulse energies, the number of BNBs increases rapidly with the laser intensity [54]. For higher energies, the number of bubbles growth decelerates and eventually reaches a plateau. This saturation in bubble nucleation can be explained by assuming that then all particles are already “activated” by the laser in the illuminated volume. In the experiments, the highly packed bubble distribution and the consequent overlapping observed in the video frames for higher energies might provoke a loss in the accuracy of the detection algorithm included in the video analysis script. As this might also play an important role in the observed saturation by imposing a limit on the measurable bubble density, a careful inspection of the results was required in those extreme cases. No bubbles are observed in control experiments where only the shock wave generator (but not the laser pulse) is fired. A small number of bubbles expand to visible size just after the laser illumination and before the shock wave pulse, as discussed in Fig. 3(b). We find that the number of bubbles already visible during laser seeding increases approximately linearly with increasing laser energy. A more extensive discussion of how particles could contribute to this behavior is detailed in Section 3.6 and Appendix B.

### 3.3 Bubble nucleation at different rarefaction amplitude and relative timing

**Fig. 5:**
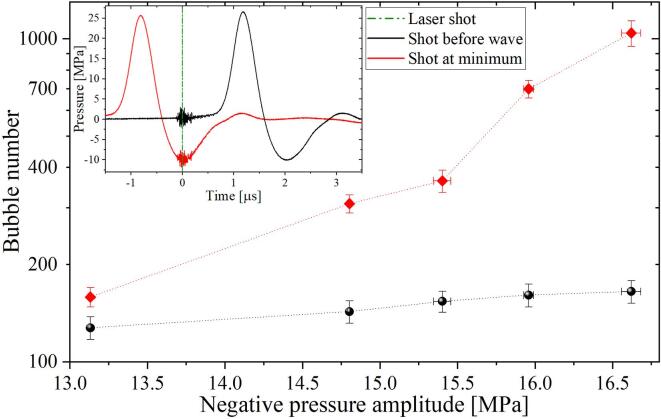
Bubble number vs. pressure amplitude. Here, the laser was fired at two different instants relative to the shock/tension wave arrival. As detailed in the inset, in one case the bubbles are seeded just before the shockwave arrival (black markers), while in the second case the laser shot was synchronized with the instant of minimum pressure in the seeding region (red markers). The laser pulse energy is approx. 9 mJ.

Here, we explore the effect of the maximum tension and the timing of the laser pulse on the resulting number of visible bubbles. The laser energy was kept constant at 9 mJ while the amplitude of the rarefaction wave was varied by changing the delay between the pressure wave arrival and the laser shot. Two series of experiments were performed. In the first series, the laser was triggered such that the nanobubbles are created during the minimum pressure amplitude of the traveling wave, see the red data points in Fig. 5 and the inset of the figure. As a result, we see a rapid increase in the number of detected bubbles for lower pressures, e.g., there are about 120 bubbles for a pressure amplitude of 13 MPa and more than 1000 bubbles for 16.5 MPa. In the second series of experiments, the nanobubbles are generated before the positive pressure pulse arrives (i.e., ∼1μs before the pressure rise shown in the inset of Fig. 5). Here, the variation in the number of bubbles is less pronounced than in the previous case, i.e., the number of nanobubbles remains at about 150 when the negative pressure is lowered. Consequently, it is quite clear that the bubble nucleation is much higher when the negative pressure is applied. This behavior could be related to a local transient change in the temperature of the liquid layer in contact with the particles, i.e., where the bubbles are produced. The connection can be established considering that an increase in the bulk temperature of the liquid is linked to a decrease in the acoustic cavitation threshold [72], [73]. Then it is likely that a similar effect occurs in the nucleation threshold of heated particles.

### 3.4 Bulk nanobubble population from dynamic Blake threshold

**Fig. 6:**
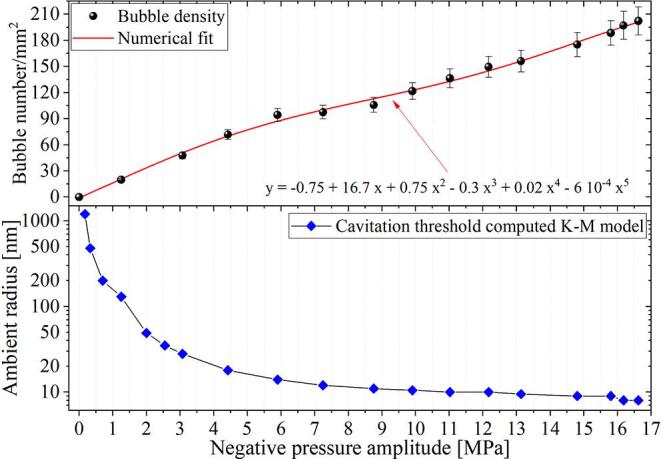
Upper panel: Bubbles density in water as a function of the acoustic pressure. Here, the energy on the laser pulse was (9 ± 0.2) mJ, and the delay between the rarefaction wave and the laser shot were set to 10 μs. Each marker represents the average of 10 individual measurements. The numerical fit performed on the experimental data (red line) allowed us to extend the results to pressure amplitudes beyond the measured (i.e., using data interpolation). Lower panel: Nucleation threshold computed with the Keller-Miksis model. The plot shows the minimum bubble size that can be expanded with a given negative pressure amplitude.

The results displayed in Fig. 5 indicate that the number of observed bubbles depends on the amplitude of the negative pressure in the wave. This is because the tension applied to the liquid containing the BNBs needs to be high enough to overcome the Laplace pressure compressing the bubble content. The minimum negative pressure required to expand a bubble of a given size is commonly referred to as *dynamic Blake threshold*. This implies that if the rarefaction wave hits a laser bubble shortly after its creation, it is possible to correlate its initial size with the negative pressure amplitude. Based on this idea we have implemented an acoustic-probe method to determine the BNBs size distribution by counting the cavities produced by rarefaction waves of increasing amplitude, as shown in Fig. 6.

**Fig. A.2:**
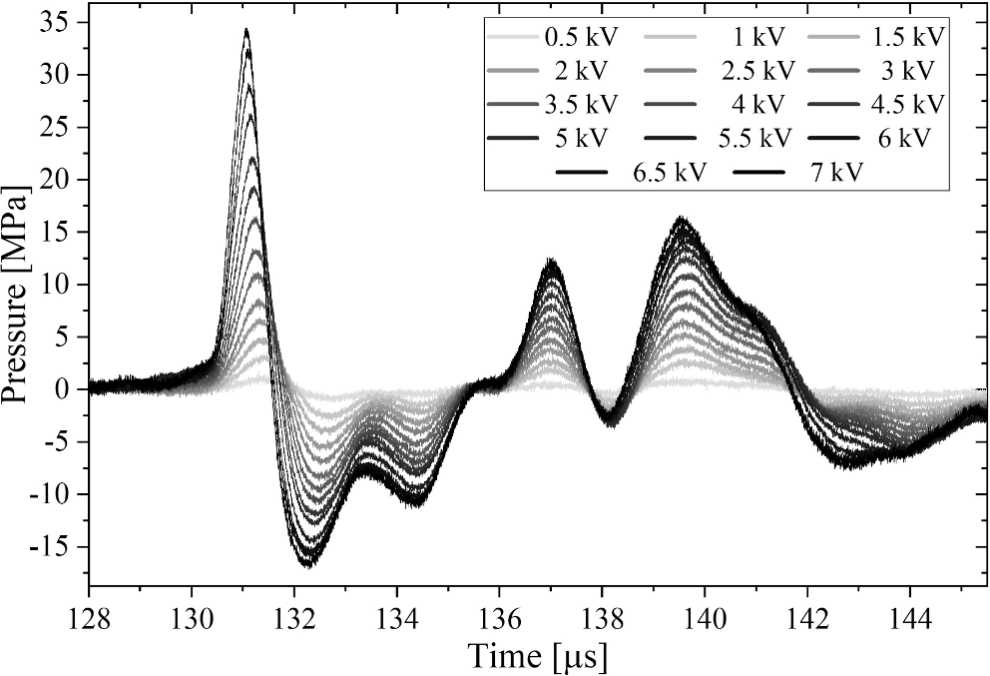
Lithotripter pressure wave for different voltages applied to the piezoceramic transducers. Each curve is an average of 20 measurements.

The upper panel of Fig. 6 gives an account of the increment on the observed bubbles as the negative pressure amplitude is raised. Here, the energy of the laser pulse was fixed to (9 ± 0.2) mJ, and the delay between the rarefaction wave and the laser shot was adjusted in 10 μs. The lower panel presents the largest bubble radius that overcomes the nucleation threshold (dynamic Blake threshold) as a function of the amplitude of the negative pressure, which is computed with the Keller-Miksis model [74], [48]. The pressure of the gas inside the bubble was modelled using the van der Waals equation of state described in Ref. [75]. Here, the hard core van der Waals radius for air was $\mathrm{hvw}=R_{0}/8.5$ [76] and the static pressure was set to 1 atm. The calculations were performed using an averaged trace of the experimental bi-polar pressure recordings, see Fig. A.2 in Appendix A. The pressure signal (and its derivative) was filtered using a Savitzky-Golay smoothing filter in order to remove the high-frequency fluctuations introduced by electronic noise. The polynomial order and frame length of the filter were changed until we found convergence of the numerical solution without distorting the physical features of the measured acoustic pressure signal.

**Fig. 7:**
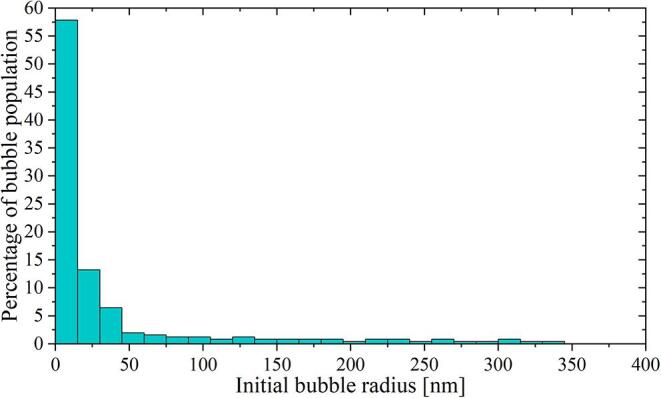
Histogram computed from the data in Fig. 6. According to this analysis, the majority of the bubbles should have a rest radius below 100 nm. The uncertainty in the bubble initial radius is around 10% for the smallest bubbles (i.e., below 100 nm) but can reach 40% for bubbles above 500 nm. The error linked to the measured bubble count rounds 4%.

The change in the bubble number was correlated with the acoustic pressure in Fig. 6. First, we computed the minimum bubble radius that could be expanded with a given negative pressure amplitude using the numerical model (indicated with diamond-shaped blue markers in the lower panel of Fig. 6). Then, we counted the change in the bubble number between two probing pressure points. Those additional bubbles should have ambient radii within the range defined by the corresponding nucleation threshold values. As an example, let us take the pressure interval between 1.5 MPa and 3 MPa. From the plot, it is possible to infer that the bubbles must have sizes comprehended between 130 nm and 30 nm. To be able to estimate the size of bubbles for pressure amplitudes between two experimental points, the measured data was fitted with a polynomial function of degree 5. In this way, the resolution of the method could be increased by interpolating the experimental results. The data correlation allowed us to compute the histogram of the bubble population presented in Fig. 7.

The histogram indicates that the totality of the bubbles is in the nanometer size range. This result is consistent with the optical limit of the system (i.e., ∼500 nm) and explains why the nanobubbles can not be detected optically. According to this analysis, most of the cavities have a radius below 100 nm. It is important to notice that there might be some gas/vapor condensation happening during the initial compression imposed by the shockwave on the nanobubbles. The uncertainty on the initial bubble radius was estimated analysing the variations found on the computed dynamic Blake threshold which, in turn, are directly linked to the fluctuations in the pressure signal measured at different iterations of the experiment.

### 3.5 Bubble population from Epstein-Plesset theory

The video frames presented in Fig. 3(b) offer an example of how BNBs behave after their generation. The bubble dynamics can be divided into two stages with their characteristic timescales. First, the rather explosive expansion of the gas nucleus is followed by a collapse cycle that typically lasts a few hundred nanoseconds. Once the bubble settles at its ambient radius, gas diffuses into the liquid at a much slower timescale under the action of the Laplace pressure [77]. This second stage can last from a few microseconds to several minutes or even hours, depending on the gas concentration in the liquid, type of gas, surface tension, and the initial rest radius of the bubble.

**Fig. 8:**
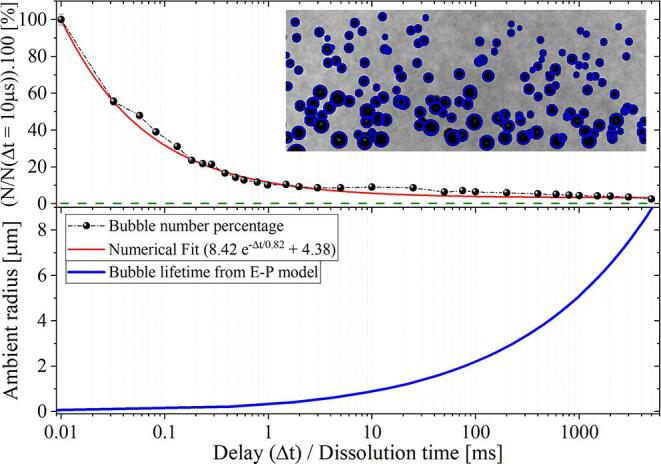
Percentage of laser-induced nanobubbles present in the water as a function of the time between seeding and observation Δt. Here, N(Δt=10μs) ≃150 (within the ROI shown in the inset). The negative amplitude of the pressure wave is (−15.0 ± 0.3) MPa. The energy on the laser pulse was (7.2 ± 0.2) mJ. Each marker represents the average of 10 individual measurements. The dashed green line indicates the bubble percentage visible immediately after the laser pulse is fired. Bottom: Bubble lifetime as a function of the initial ambient radius computed for water at ambient temperature using the Epstein-Plesset model.

In order to expand the nanobubbles and make them visible, the rarefaction wave must reach the gas cavities before shrinking below their dynamic Blake threshold. As a matter of fact, the duration for full dissolution from the threshold radius is rather short (of the order of hundreds of nanoseconds). Therefore, we simplify the problem and neglect that very last part when measuring the lifetime of the bubbles. Thus the permanence of the nanobubbles in the liquid can be tested by counting the remaining number of bubbles in the solution after a given delay (Δt) between the seeding laser pulse and the tension wave arrival. With that in mind, we performed an experiment varying Δt while keeping both the acoustic amplitude and the laser pulse energy fixed to (−15.0 ± 0.3) MPa and (7.2 ± 0.2) mJ, respectively. The results shown in Fig. 8 demonstrate that the bubble population decays rapidly, i.e., within 1 ms after the seeding takes place. The relatively short lifetime of the bubbles allows for neglecting gravity or interaction between bubbles such as collisions and/or merging.

**Fig. 9:**
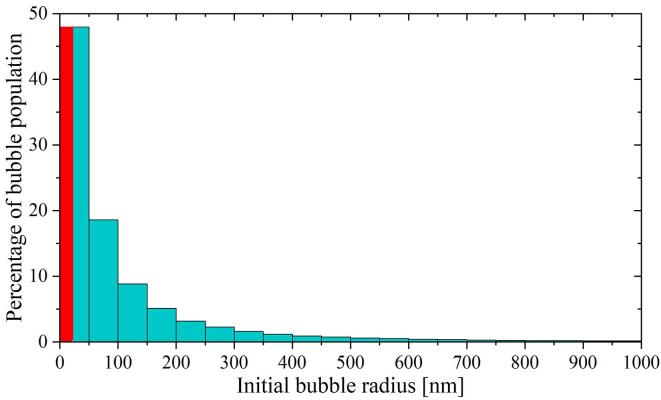
Histogram of the bubble population computed from the dissolution times displayed in Fig. 8. Here, the decay on the bubble number observed for increasing Δt was correlated with the initial size of the bubbles through the Epstein-Plesset theory. The red bar indicates the values that are beyond the computational limit imposed by the minimum delay Δt set in the experiments. The uncertainty linked to the measured bubble count rounds to 4%.

The dissolution dynamics of the bubbles are modeled with the Epstein-Plesset equation [77]. The lower panel of Fig. 8 presents a calculation of the bubble lifetime corresponding to bubbles in water with an ambient radius ranging from 20 nm to 9 μm. Let us now correlate the bubble count observed in the experiments for different delays Δt with the dissolution time computed from the Epstein-Plesset theory. The method here is similar to the probing of the dynamic Blake threshold introduced in Section 3.4. The outcome of such a correlation is shown in the histogram of Fig. 9. The bubble size distribution obtained by this method confirms the nanometric size of the bubbles. As a result, 75% or more of the bubbles initially produced by the laser have a radius below 200 nm while the remaining 25% of the bubbles do not exceed 500 nm.

### 3.6 Stability and characteristics of bulk nanobubbles in a NaCl salt solution

The bubble size analysis method based on the Epstein-Plesset theory can be also applied to study the diffusive stability and characteristics of bulk nanobubbles for a wide variety of liquids. As a first test, we use two aqueous solutions of sodium chloride (NaCl) dissolved in nanopore water with concentrations of 4% *w/w* (i.e., 0.68 *mol*/l, slightly above the seawater salt concentration) and 10% *w/w* (1.71 *mol*/l). The main differences between the clean water and the NaCl samples were noted in the acoustic and laser nucleation thresholds. The acoustic cavitation threshold of the salty samples was lower than the one in water. As a consequence, the amplitude of the rarefaction wave has to be reduced in all cases to prevent cavitation produced by wave passage. Additionally, the laser bubble seeding rate was much higher in the cases with added NaCl. In order to have samples with similar bubble populations as in the nanopore water the laser energy was lowered to match the initial number of bubbles found in the pure water sample. These adjustments aimed to avoid an excessive overlapping of bubbles which might saturate the video frames, preventing accurate bubble detection.

**Fig. 10:**
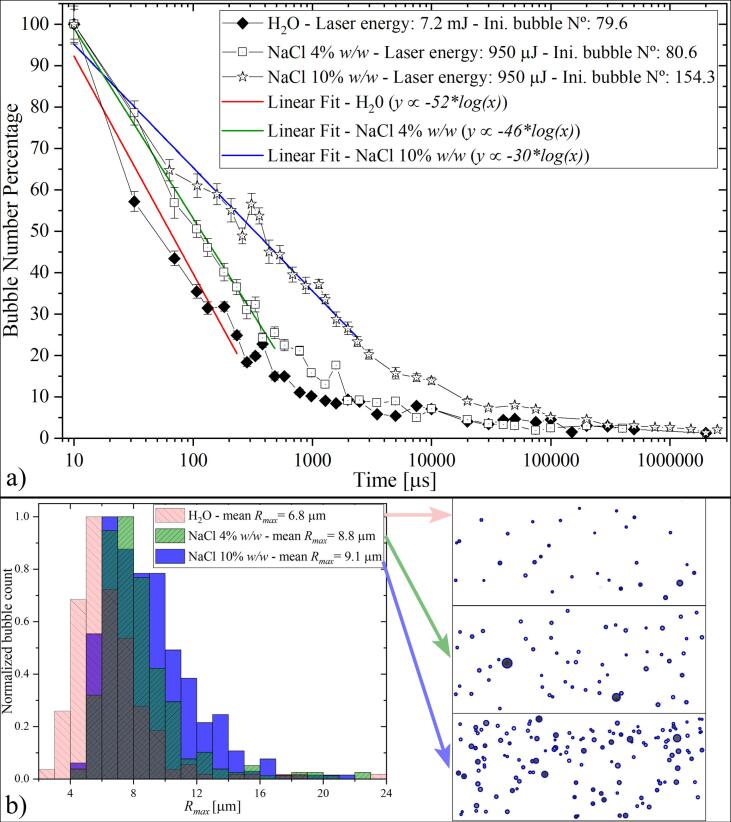
Nanobubble dissolution in samples with added NaCl. The negative amplitude of the pressure wave is (−5.8 ± 0.3) MPa. (a) Bubble lifetime. The initial number of bubbles (i.e., at Δt = 10 μs) was adjusted by reducing the laser pulse energy in the salty samples. The lines are used as a visual aid to highlight the lower decay rate with increasing concentrations of NaCl. (b) Bubble population immediately after the laser shot (i.e., before the rarefaction wave arrival). Here, the energy of the laser pulse was the same for the three samples (∼11 mJ). The bubble maximum size attained during the laser nucleation was obtained from the video frames (see insets).

**Fig. B.1:**
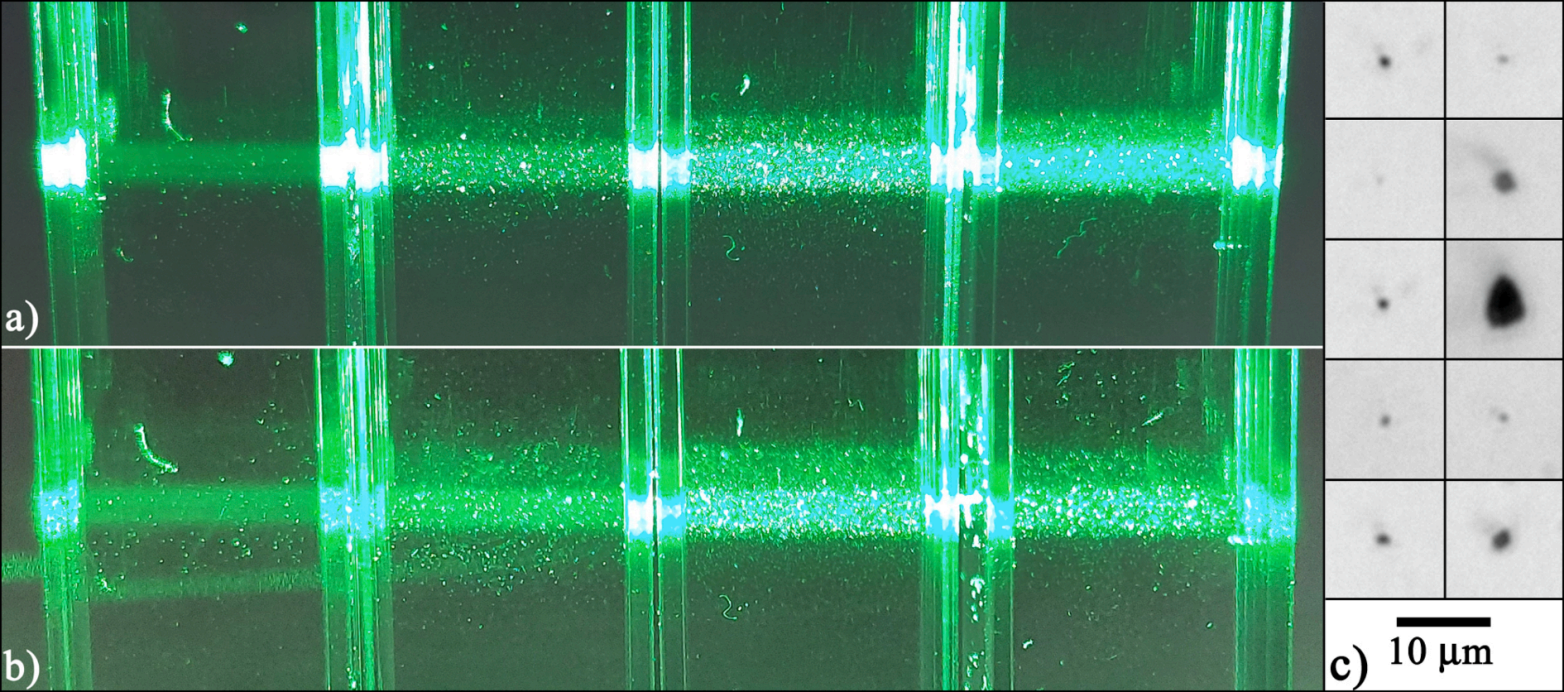
Visualization of the Tyndall effect on the liquid samples. From left to right the cuvettes contain nanopore water, NaCl solution at 4% *w/w* (i.e., 0.68 *mol*/l), NaCl solution at 10% *w/w* (1.71 *mol*/l), and nanopore water after 15 min of sonication treatment. a) Here the sonicated sample is shown 30  min after sonication. b) In this picture the sonicated sample was frozen for 3 h and later unfrozen. No significant difference in the scattered intensity was found. c) Titanium flakes found in the sonicated sample. These micrometer-sized particles originate from cavitation erosion of the sonotrode tip.

The resulting curves for the decay in the bubble population of the three samples are shown in Fig. 10. Fig. 10(a) reveals a mild reduction in the dissolution rate of the NaCl 4% solution and a slower rate for the higher salt concentration (NaCl 10%). This can be explained by the reduction of the diffusion coefficient and the air solubility of water when NaCl in added to the liquid [78], [79], [80], [81]. We have also observed a larger number of bubbles nucleated with the rarefaction wave and the laser pulse. This may be caused by insoluble impurities present in the NaCl powder. Even analytical grade monovalent salts may contain a small portion of mineral and metallic particles, which is evident from light scattering, see Fig. B.1 in Appendix B. As discussed in the previous sections, the laser pulse by its own can nucleate visible cavitation bubbles (e.g., Fig. 3b) and the green line in Fig. 4) even for lower laser pulse energies. In the experiments using salty water, this phenomenon is more pronounced due to a higher presence of particles in the liquid, as illustrated in Fig. 10(b). There, the laser pulse intensity was set to the maximum for the three samples to promote the bubble nucleation and thus produce enough data points to perform a statistical study. The size achieved by the laser-induced bubbles immediately after their formation was characterized using the automated detection algorithm, see insets in Fig. 10(b). The images make evident the proportionality between the bubble number and the salt concentration. Moreover, the histograms on the left panel of Fig. 10(b) indicate that the bubbles growing from the particles in the salt solutions are bigger than the ones produced in water, which indirectly means that the particles in the liquid are probably bigger, as discussed on Appendix B.

**Fig. 11:**
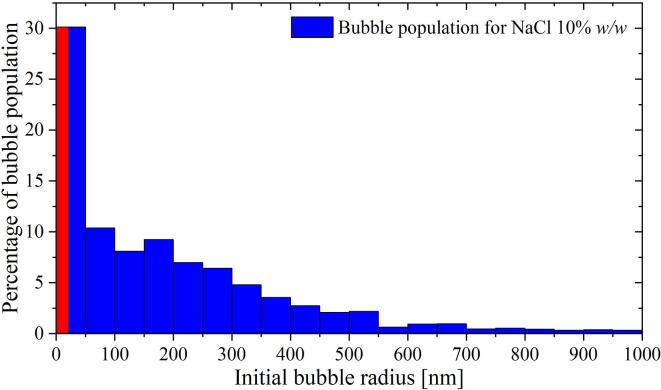
Histogram of the bubble population computed from the dissolution times depicted in Fig. 10 for aqueous NaCl solution of 10% *w/w*. Here, the bubble population is shifted to slightly larger radii as compared to the nanopore water. The uncertainty in the diffusion coefficient and solubility of the salt solution propagates as an error of 16% on the computed bubble lifetime. The error linked to the measured bubble count rounds to 4%.

The initial bubble size of the sample with the NaCl solution at 10% *w/w* was also quantified following the Epstein-Plesset method described in Section 3.5. The results shown in Fig. 11 corroborate the previous assumptions on the increased bubble size of the samples with NaCl. However, it is important to remark that the values obtained by this method might be less accurate due to an uncertainty of at least 15% in the physical properties used for the calculations. At the same time, the suitability of the Epstein-Plesset equation for modeling the dissolution of nanobubbles in solutions with high concentrations of ions like salts, acids or alkaline substances is still under debate, as discussed in the introduction.

Recent research on BNBs produced in water containing dissolved salts reports that the bubbles are stabilized at a larger mean radius as compared to BNBs in distilled water [9], [30], [5], [8], [7]. Their larger mean radius seems to reduce however the long-term stability of the BNB solution[8], i.e., over many days. On shorter timescales, the coalescence of microbubbles is prevented in aqueous salt solutions[61], [62].

## 4 Conclusions

We have extended our previously published method for the detection of laser-induced bulk nanobubbles (BNBs) by using a separate shock wave generator and automating the generation and detection process. In this way, repeatable measurements of the BNBs allowed the measurement of their size and distribution. Here, two distinct methods result in comparable distributions, namely based on their dissolution dynamics and their response to increasing rarefaction wave amplitudes. These techniques may accompany and extend sizing techniques that are based on laser light scattering, such as dynamic light scattering and nanoparticle tracking.

Overall, we find a heterogeneous distribution of nanobubbles even when using clean MilliQ water passed through 50 nm pore-size filters. The observed nucleation of bubbles on suspended particles in tap water points towards a mechanism that the nanobubbles are nucleated by absorption and the successive heating of the water in contact. The necessary seed particles may be unresolved impurities present in the samples or colloidal particles added to the liquid sample. Thus the use of the term “clean” to define the current approach seems contradictory considering that the presence of particles is an essential aspect of nanobubble generation. However, the term refers to the simple fact that no particles are introduced during the BNB production, as opposed to standard nanobubble generators that utilize moving parts in contact with the liquid.

The stability of the bubble clusters was studied directly by measuring the lifetime of the bubbles and not the stability of the colloidal system based on laser scattering. This approach thus constitutes an important step towards a better understanding of this matter. So far we haven’t found evidence of the formation of long-lived bulk nanobubbles in any of the analyzed samples. This does not mean that they can not be produced under different conditions beyond the ones explored in this study. This promising technique could be used to further explore a wide variety of experimental scenarios; for instance, by varying the laser pulse characteristics, using sample liquids with different properties or dissolved concentrations of gases, and by changing the liquid temperature or the static pressure. Additionally, the systematic addition of colloidal particles of various sizes and materials to the liquid samples could lead to a better comprehension of the physics of nanobubble nucleation.

## CRediT authorship contribution statement

**Juan Manuel Rosselló:** Conceptualization, Methodology, Data curation, Formal analysis, Software, Visualization, Investigation, Writing - original draft. **Claus-Dieter Ohl:** Conceptualization, Resources, Methodology, Writing - review & editing.

## Declaration of Competing Interest

The authors declare that they have no known competing financial interests or personal relationships that could have appeared to influence the work reported in this paper.

## Figures and Tables

**Fig. A.1:**
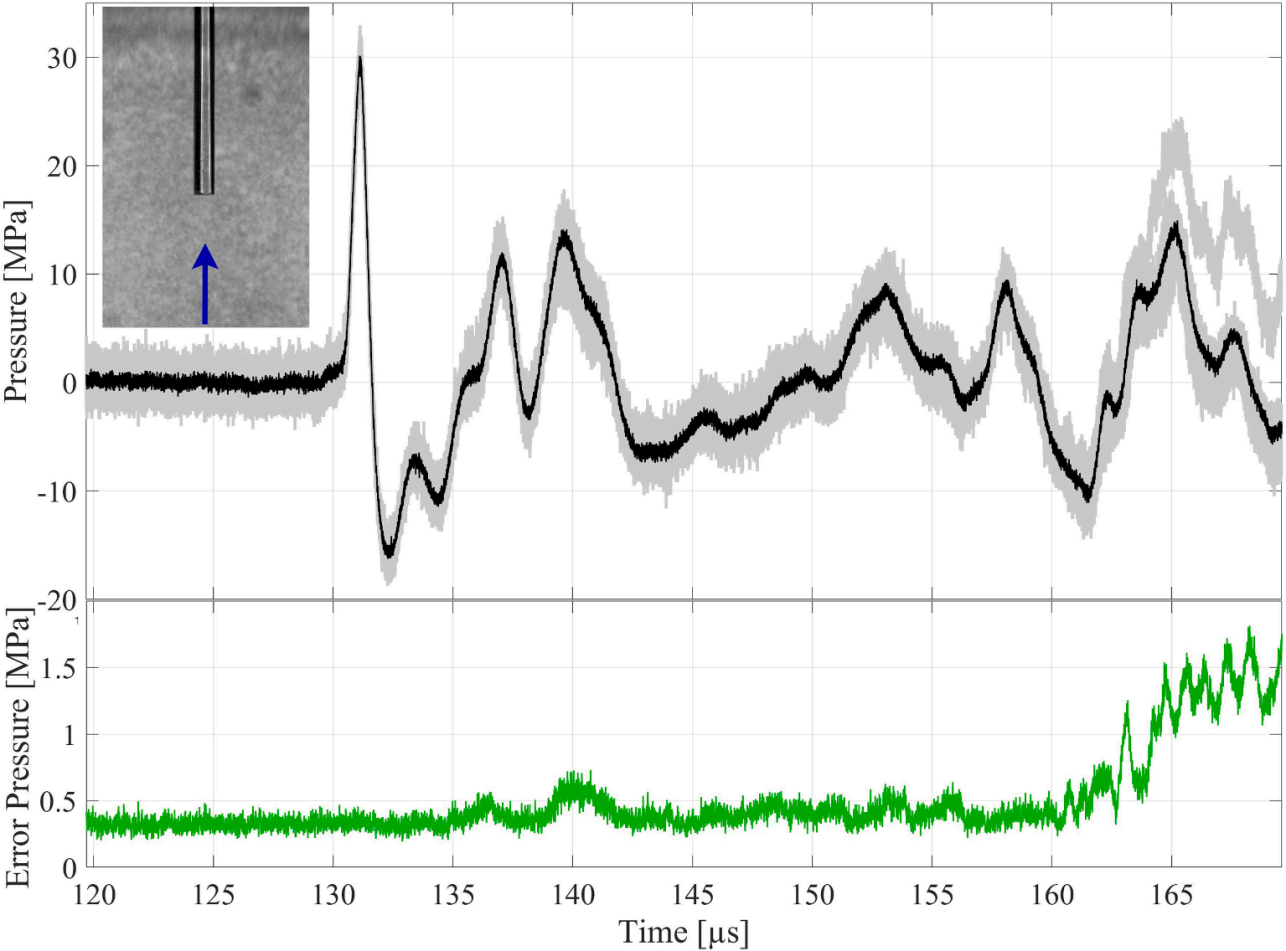
Lithotripter pressure wave. (a) Example of the bipolar signal measured at its focal point. The image shows an overlapping of 10 consecutive pressure waves in grey and the averaged signal in black. The initial time (t=0) was given by the instant when the trigger signal is applied to the lithotripter. The inset shows the fiber optic hydrophone tip used in the measurements and the blue arrow indicates the direction of the incoming shock wave. (b) Statistical error of the averaged shock/tension wave. The error associated to the measurement on the bi-polar pressure wave driving the nanobubbles is below 0.5 MPa, giving account on the high repeatability of the signal. The increased dispersion of the samples observed after *t* = 160 μs is produced by the collapse of cavitation bubbles nucleated by the rarefaction wave in the vicinity of the hydrophone tip.

**Fig. B.2:**
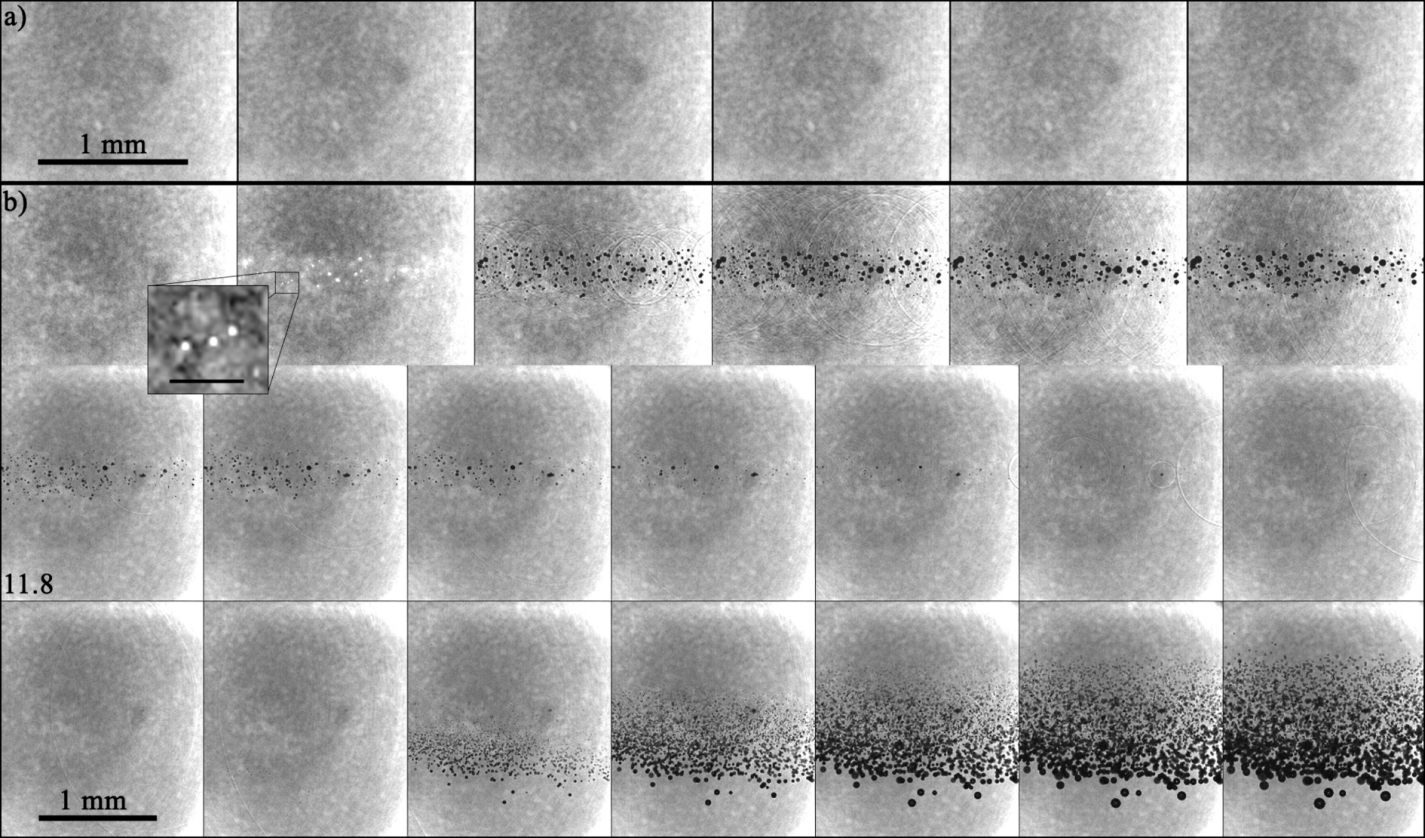
Bubble nucleation on nanopore water after sonication. The amplitude of the pressure wave was (32.0 ± 0.5) MPa. The energy on the laser pulse was (3.0 ± 0.2) mJ. The time between frames is 200 ns. a) No bubbles were nucleated by acoustic cavitation (without firing the laser). b) When the laser is fired many bubbles are observed immediately after the exposure to the light, i.e., before the arrival of the rarefaction wave. The inset shows heterogeneous light scattering spots (the bar represents 100 μm). This is a clear indicator of the presence of laser energy-absorbing particles. The last two rows of the image sequence show the instant where the bipolar acoustic wave arrives.

**Fig. B.3:**
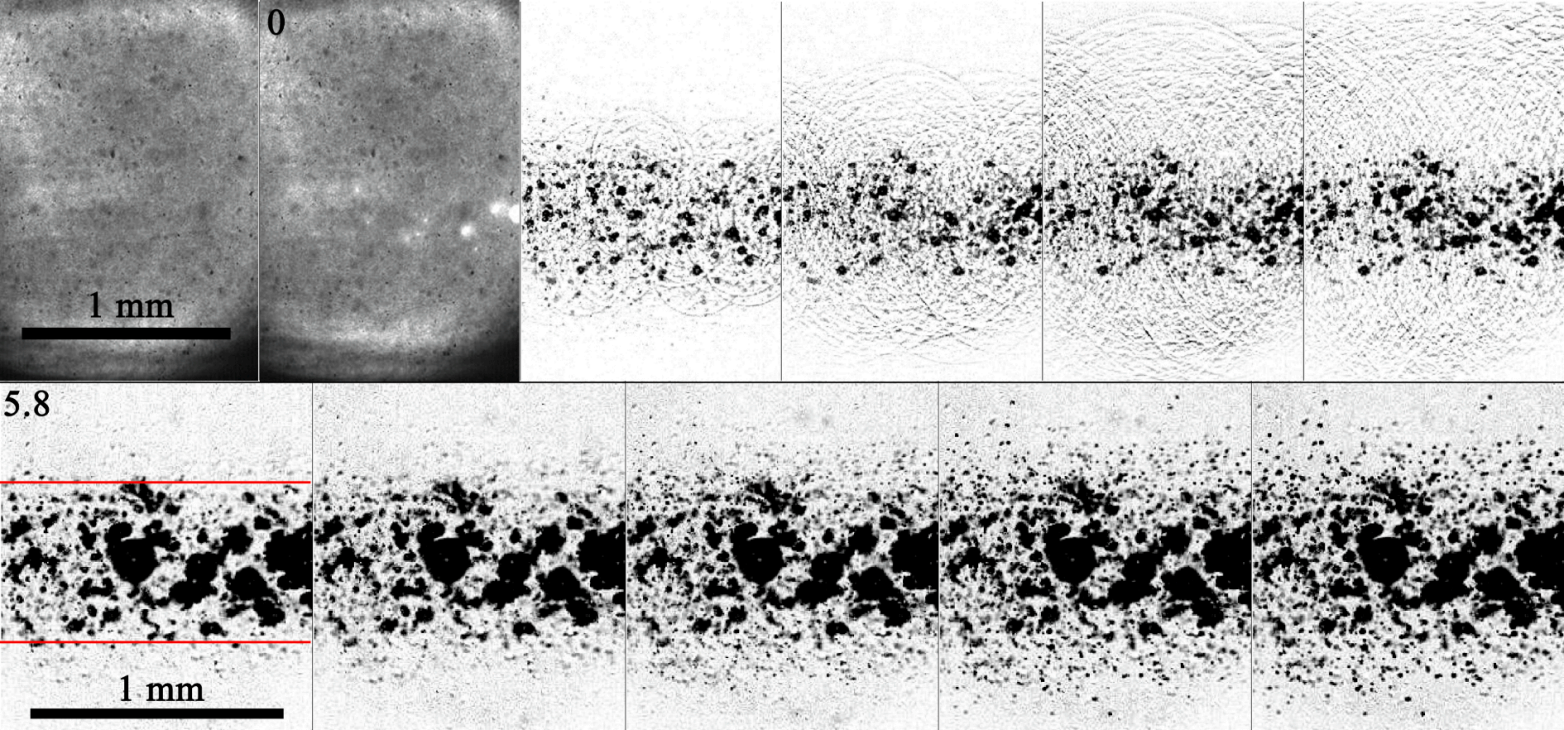
Laser bubble nucleation on the water with graphite particles in solution (with a mean size of ∼8 μm). Here, the bubbles are nucleated only by laser absorption and no external acoustic wave was applied. The energy on the laser pulse was (11.2 ± 0.5) mJ. The time between frames is 200 ns for the upper row and 400 ns for the lower row. The numbers indicate time in microseconds. When the laser is fired the number and the size of the bubbles produced by light absorption is remarkably high. This gives origin to a wavefront formed by the superposition of thousands of shockwaves emitted by the bubbles, as shown after the third frame, where the background of the images has been subtracted. The last row shows how the initially invisible bubbles located in the periphery of the Gaussian laser beam (marked with a red line) are expanded by the sound soft reflections of the shockwaves on the expanded cavities and the cuvette walls.

## Appendix A Characterization of the shock/tension wave

A precise characterization of the bi-polar pressure wave generated by the lithotripter-type shock wave generator is a key factor for making a correct interpretation of the experimental data, and also a necessary step in order to perform realistic numerical simulations. Accordingly, we used a fiber optic hydrophone *Onda HFO-690* with a spatial resolution of 100 μm to measure the pressure wave emitted by the lithotripter at its focal spot for a range of voltages set on the piezoceramic elements on the device (PZT). These fast-varying signals were stored with a sampling rate of 20 GSa/s (analog bandwidth 4 GHz). The use of this kind of small and smooth sensor allowed us to measure the rarefaction waves without the typical distortions produced by the acoustic nucleation of bubbles in the hydrophone tip. To further prevent the bubble formation we added acetic acid to the nanopore water up to a concentration of 4% *w/w*, following Refs. [82], [83].

An exemplary curve of the pressure signal averaged from 10 consecutive measurements is presented in Fig. A.1 for a case with a positive pressure amplitude of ∼28 MPa, produced by applying 6 kV to the piezoelectric ceramics of the lithotripter. The data set corresponding to this figure can be found in the Supplementary material. The signal is remarkably repeatable, in particular, in the first few microseconds where the initial bipolar wavefront occurs. The low degree of dispersion observed in the rarefaction wave (happening between t≃131 μs and t≃135 μs in Fig. A.1 allowed us to correlate the number of bubbles observed (after their expansion) with their rest radius without the need of measuring the signal on every laser shot, as described in Section 3.3.

Another interesting feature of this shockwave generator is that the shape of the acoustic signal is conserved for the whole range of acoustic pressures used in this work, as shown in Fig. A.2. This means that the bubbles are driven by the same kind of pressure signal for all the amplitudes used. This is very relevant since the expansion of the bubbles is sensitive to small fluctuations in the pressure signal. Additionally, numerical scaling would be possible, at least for acoustic signals located between two measured curves.

## Appendix B Particle contamination and its role in the bubble production

One of the main motivations of this study is to provide an alternative nanobubble generation method that overcomes the issues related to sample contamination. In this Appendix, we present experimental evidence of a case in which a sample of nanopore water is sonicated following, with the outcome of the sample contamination with titanium particles coming from the sonotrode tip [38], [39]. Additionally, we show how the presence of laser-light-absorbing particles in the sample changes dramatically the size and the number of bubbles nucleated by the laser.

The sonicated samples were prepared with a sonotrode *Bandelin Sonopuls Ultrasonics Homogenizer UW 2200* by putting nanopore water in a standard 100 ml beaker and then submerging the titanium tip of the sonotrode (*MS72*) 3 cm into the liquid. The beaker with the sample was placed in a water-circulating bath to keep the liquid at a room temperature of 22 °C. Afterward, the cubicle containing the sample and the sonotrode is closed to avoid any external contamination. The liquid is sonicated by a sinusoidal driving signal with a frequency of 20 kHz delivering a power of 36 W in intermittent cycles of 500 ms for 15 min. After sonication, the sample was stored in a clean and closed bottle.

As shown in Fig. B.1, when illuminated with a continuous wave green laser (1 W of power) the sonicated sample scatters an increased amount of light (i.e., Tyndall effect) as compared to a non-treated water [84], [6].

The formation of nanobubbles in the sonicated liquid was tested following the procedure explained in Section 2 of the main text. Accordingly, the sample was placed in the tailored cuvette shown in Fig. 1 and probed with a shock/tension wave at the maximum amplitude of around (32 ± 0.5) MPa. (i.e., approximately −16 MPa of negative pressure). In this case, no significant number of expanded bubbles were observed (see Fig. B.2(a)). Immediately afterward we repeated the operation but this time by illuminating the liquid with a collimated high-power laser pulse of around 3 mJ. In this second attempt, a huge amount of bubbles were noticed even during the laser beam irradiation (much before the rarefaction wave arrival) as shown in Fig. B.2(b). The visualization of bright spots indicates the presence of particles scattering the laser light while the bubbles preceding the laser shot demonstrate a significant laser energy absorption, being both factors a clear sign of the existence of solid particles in the sample. The presence of contamination in the form of titanium particles of around 2 μm deposited in the liquid was corroborated using a microscope (see Fig. B.1(c)). Those were produced by cavitation erosion during sonication. The particles had the shape of a flake and move continuously in the sample following a Brownian motion pattern. The samples with added NaCl shown in Fig. B.1 also exhibit a higher intensity in the scattered light as the solute concentration is increased. In this case, the particles might come from the 0.5% of metallic traces reported by the salt supplier as potential impurities. No particles were observed under the microscope in the nanopore water.

A second example of how particles in solution can nucleate bubbles by absorbing laser energy is presented in Fig. B.3. There graphite particles with a mean size of ∼8 μm were added to nanopore water in a concentration of around 4 g/l. The graphite powder particles have the shape of a flake with typical sizes ranging from 2 μm to 50 μm. In this case, the amount and the size of the laser-induced bubbles were so big that an explosive Huygens–Fresnel-like wavefront was emitted from the region containing the laser beam. Here, the application of a rarefaction wave to the heated particles would instantly saturate the camera frame with bubbles, thus no external acoustic signals were applied in this case.

These experimental results are consistent with the previously observed four cases with smaller particles. The strong dependence of the number of bubbles observed immediately after the laser illumination and their correlation with the particle size supports the hypothesis of nanobubbles generated from the absorption of laser energy by nanoscopic particles.
